# Validation of a guideline to reduce variability in diagnosing cervical dystonia

**DOI:** 10.1007/s00415-023-11585-6

**Published:** 2023-02-15

**Authors:** Giovanni Defazio, Daniele Belvisi, Cynthia Comella, Mark Hallett, Hyder A. Jinnah, Paola Cimino, Anna Latorre, Marcello Mario Mascia, Lorenzo Rocchi, Angelo Fabio Gigante, Tommaso Ercoli, Alfredo Berardelli

**Affiliations:** 1 Department of Medical Sciences and Public Health, Institute of Neurology, University of Cagliari, 09042 Cagliari, Italy; 2 Institute of Neurology, Azienda Ospedaliero Universitaria di Cagliari, SS 554 km 4.500, 09042 Monserrato, Cagliari Italy; 3 IRCCS Neuromed, Via Atinense 18, 86077 Pozzilli, IS Italy; 4 Department of Human Neurosciences, Sapienza University of Rome, Viale dell’ Università, 30, 00185 Rome, Italy; 5 Rush University Medical Center, New Philadelphia, OH USA; 6 Human Motor Control Section, NINDS, NIH, Bethesda, MD USA; 7 Department of Neurology and Human Genetics, Emory University, Atlanta, GA USA; 8 Department of Clinical and Movement Neurosciences UCL Queen Square Institute of Neurology, University College London, London, UK; 9 Section of Neurology, San Paolo Hospital, Bari, Italy

**Keywords:** Cervical dystonia, Guideline, Diagnosis

## Abstract

**Background:**

Cervical dystonia is characterized by a variable pattern of neck muscle involvement. Due to the lack of a diagnostic test, cervical dystonia diagnosis is based on clinical examination and is therefore subjective. The present work was designed to provide practical guidance for clinicians in confirming or refuting suspected cervical dystonia.

**Methods:**

Participants were video recorded according to a standardized protocol to assess 6 main clinical features possibly contributing to cervical dystonia diagnosis: presence of repetitive, patterned head/neck movements/postures inducing head/neck deviation from neutral position (item 1); sensory trick (item 2); and red flags related to conditions mimicking dystonia that should be absent in dystonia (items 3–6). Inter-/intra-rater agreement among three independent raters was assessed by *k* statistics. To estimate sensitivity and specificity, the gold standard was cervical dystonia diagnosis reviewed at each site by independent senior neurologists.

**Results:**

The validation sample included 43 idiopathic cervical dystonia patients and 41 control subjects (12 normal subjects, 6 patients with isolated head tremor, 4 with chorea, 6 with tics, 4 with head ptosis due to myasthenia or amyotrophic lateral sclerosis, 7 with orthopedic/rheumatologic neck diseases, and 2 with ocular torticollis). The best combination of sensitivity and specificity was observed considering all the items except for an item related to capability to voluntarily suppress spasms (sensitivity: 96.1%; specificity: 81%).

**Conclusions:**

An accurate diagnosis of cervical dystonia can be achieved if, in addition to the core motor features, we also consider some clinical features related to dystonia mimics that should be absent in dystonia.

**Supplementary Information:**

The online version contains supplementary material available at 10.1007/s00415-023-11585-6.

## Introduction

According to the most recent consensus update [1], dystonia is defined as a condition characterized by “sustained or intermittent muscle contractions causing abnormal, often repetitive, movements, postures, or both. Dystonic movements are typically patterned, twisting, and may be tremulous. Dystonia is often initiated or worsened by voluntary action and associated with overflow muscle activation” [1].

Cervical dystonia (CD), the most frequent form of focal dystonia, is characterized by a variable pattern of neck muscle involvement, leading to clinically heterogeneous directional presentations, such as torticollis, laterocollis, retrocollis, or anterocollis [2]. Patients may also have additional signs and symptoms, including shoulder elevation, neck/shoulder pain, or head tremor, and may benefit from the use of sensory tricks, a highly specific maneuver that may induce transient amelioration of dystonia [3–5]. In some patients dystonic activity may also spread to other body parts [6–9].

Due to the lack of a diagnostic test, CD diagnosis is based on clinical examination and is therefore subjective [10]. As an example, a study on CD incidence in northern California found that up to 65% of patients may be incorrectly diagnosed prior to receiving a correct diagnosis [11]. Further support of a high rate of CD underdiagnosis as a ubiquitous phenomenon also derives from family-based studies from studies assessing diagnostic delay in several geographic areas [12–16]. Diagnostic errors may largely be due to the clinical variability of CD but also to the existence of several related conditions, for example, pseudodystonia mimicking the abnormal movements or postures of CD [17, 18]. In the case of CD, dystonia mimics may include neck chorea producing non-repetitive head movements [19]; neck tics associated with ability to mentally suppress the spasms [20]; orthopedic neck diseases (like atlanto-axial and shoulder subluxation, or fracture of the cervical vertebrae), rheumatologic neck diseases, and posterior fossa tumors, all leading to tonic postures or movement of the head [21]; lower motor neuron disease/myopathy/myasthenia gravis inducing weakness of the neck muscles opposite to the abnormal posture [22, 23]; and ocular torticollis characterized by diplopia caused by the voluntary correction of the abnormal neck posture [24].

According to expert opinion [25], the clinical diagnosis of CD should rely on the core motor features highlighted in the revised definition of dystonia [1], and the exclusion of clinical red flags related to neurological/non-neurological conditions mimicking dystonia (that would be expected to be absent in dystonia) [25]. Whether these key features may help diagnose CD and differentiate the condition from other disorders of the neck that resemble dystonia has never been assessed in terms of diagnostic sensitivity and specificity. The present work was designed in the attempt to minimize sources of diagnostic errors and to provide practical guidance for clinicians in confirming or refuting suspected CD.

## Methods

Participants were identified from among outpatients attending the movement disorder clinic of the University of Cagliari and Sapienza University of Rome. Inclusion criteria for both case and control subjects were age 18 or older, any sex, and the willingness and mental/physical ability to sign informed consent and participate in the protocol. Case patients were enrolled if they had a diagnosis of focal idiopathic CD made by an experienced movement disorder neurologist [25, 26]. Exclusion criteria were secondary CD and co-existing medical conditions/surgical interventions that could confound assessment of CD. Botulinum neurotoxin (BoNT) treatment was performed at least 12 weeks before the examination. The control group included normal subjects and a group of patients with head/neck impairment that could be confused with CD [18], i.e., isolated head tremor; non-repetitive head movements due to chorea; head tics associated with the ability to mentally suppress spasms; fixed involuntary neck postures due to orthopedic neck diseases (like atlanto-axial and shoulder subluxation or cervical vertebrae fracture), rheumatologic neck diseases, or posterior fossa tumors; focal weakness of the neck muscles opposite the side of abnormal posture due to lower motor neuron disease/myopathy/myasthenia gravis; and diplopia caused by the voluntary correction of abnormal neck posture due to ocular torticollis (Supplemental Table 1).

To assess diagnostic accuracy we focused on the following clinical items: (i) presence of repetitive, patterned head/neck movements/postures inducing head/neck deviation from neutral position (item 1, derived from the 2013 revised definition of dystonia) [1]; (ii) sensory trick (item 2); and (iii) red flags related to conditions mimicking dystonia that would be expected to be absent in dystonia (items 3 to 6). In the latter group, we took into account fixed head/neck deviation from neutral position (item 3, a feature distinguishing dystonia from orthopedic or rheumatologic diseases inducing fixed postures); focal weakness of neck muscles antagonizing the abnormal head/neck posture (item 4, a feature that may prove useful to differentiate lower motor neuron diseases/myopathy from dystonia); diplopia induced by voluntary correction of the abnormal head/neck posture (item 5, a feature that may distinguish CD from ocular torticollis); and ability to voluntarily suppress spasms defined as an inner volitional effort rather than voluntary compensatory frontalis muscle overactivity (item 6, a feature that is potentially useful to distinguish dystonia and tics). Attention was paid to distinguish suppressibility by willpower alone from compensatory movements that often counteract dystonic movements or postures and are also the result of voluntary action. There was no duration requirement for voluntary suppression.

Participants were video recorded according to a standardized protocol in order to assess all the major/distinctive clinical features possibly contributing to CD diagnosis. The video protocol included standard maneuvers triggering involuntary head movements, sensory trick if present, and the strength of neck muscles under voluntary contraction. Patients were asked to demonstrate their trick to the examiner. Tricks were predominantly tactile and included touching the chin, cheek, or neck; in two patients, visual fixation temporarily improved CD. Trick was considered to be effective when it induced complete cessation or at least some decrease in dystonic position. In several patients, sensory trick was effective when applied on the side contralateral to the dystonic movement. In other patients who applied the trick on the same side of the dystonic movement, counterpressure was excluded if the patient employed only a mild force to counteract the dystonic contraction. Subjects were also asked by the examiner about: (i) occurrence of diplopia induced by voluntary correction of the abnormal head/neck posture and (ii) capability to voluntarily suppress involuntary neck movements.

Inter-/intra-rater agreement was assessed among three independent raters who did not belong to the centers participating in the project. The number of videos included in the reliability study (64 video recordings of 43 CD patients, 6 normal controls, and 15 disease controls) exceeded that based on recommended subject-to-item ratios (which usually consider the assessment of 5–10 subjects for each item of a new scale) and on the number of items (*n* = 4) to be assessed by the three observers. Item 5 (diplopia induced by voluntary correction of the abnormal head/neck posture) and item 6 (inability to voluntarily suppress spasms) were not included in the reliability analysis because questions about these items were asked by the site examiner but not captured in the video. Agreement among raters was assessed by *k* index, which measures the level of agreement beyond chance and ranges from − 1 (perfect disagreement) to + 1 (perfect agreement). A *k* index > 0.4 (indicating moderate to substantial/almost perfect agreement) was considered to be satisfactory.

To estimate sensitivity and specificity, the gold standard was the diagnosis made at each site by the senior neurologists (GD and AB). Sensitivity was defined as the proportion of subjects who screened positive from among those who had a diagnosis of CD on clinical examination (true positives/true positives + false negatives). Specificity was the proportion of subjects who screened negative from among those who were determined to not have CD on clinical examination (true negatives/false positives + true negatives).

This study was approved by the ethic committee (identification no. PG/2018/7281). Written informed consent was obtained from all participants. A signed patient consent-to-disclose form was obtained for videos of any recognizable patient.

## Results

The validation sample included 43 patients with idiopathic CD (age at onset 53.3 ± 9.5 years) and 41 control subjects. The control group included 12 normal subjects, 6 patients with isolated head tremor, 4 with chorea, 6 with tics, 4 with head ptosis due to myasthenia or amyotrophic lateral sclerosis, 7 with orthopedic/rheumatologic neck diseases, and 2 with ocular torticollis. The case and control groups were similar for sex (29 women and 14 men vs. 27 women and 14 men, *p* = 0.5) and age (60.8 ± 10.7 vs. 59.5 ± 12.7 years, *p* = 0.4).

Inter-rater agreement was substantial to almost perfect for all four tested items (item 1: *k* = 0.82, *p* < 0.0001; item 2: *k* = 0.87, *p* < 0.0001; item 3: *k* = 1.00, *p* < 0.0001; item 4: *k* = 0.86, *p* < 0.0001).

Considering only item 1 (“stereotyped, patterned, involuntary head/neck movements or postures inducing head/neck deviation from neutral position”), the three observers achieved 98% mean sensitivity and 48% mean specificity (Table 1); analyzing item 2 alone (i.e., sensory trick), mean sensitivity was 75% and mean specificity was 84%; finally, mean sensitivity and mean specificity of the red flags group (namely, items 3–6) were 55 and 71%, respectively (Table 1).

**Table 1:** Sensitivity and specificity of clinical diagnostic items 1, 2, and 3 to 6

	Sensitivity			Specificity			Average sensitivity (%)	Average specificity (%)
	Observer 1	Observer 2	Observer 3	Observer 1	Observer 2	Observer 3		
Repetitive, patterned head/neck movements/postures inducing head/neck deviation from neutral position (item 1)	100% (43/43)	97.6% (42/43)	95.3% (41/43)	42.5% (17/41)	51.2% (21/41)	51.2% (21/41)	97.6	48.3
Sensory trick (item 2)	74.4% (32/43)	74.4% (32/43)	76.7% (33/43)	80.5% (33/41)	80.5% (33/41)	90.2% (37/41)	75.1	83.7
Red flags related to conditions mimicking dystonia (items 3–6)	58% (25/43)	51% (22/43)	53.5% (23/43)	80.5% (33/41)	71.4% (29/41)	60.9% (25/41)	54.7	70.9

Owing to the unsatisfactory levels of sensitivity and specificity, we tested whether combining the selected items would improve diagnostic sensitivity/specificity (Table 2). First, we combined the item that reached the greatest sensitivity, that is item 1, with item 2, or the red flags group (items 3–6): the combination item 1 + item 2 yielded 74% mean sensitivity and 89% mean specificity (Table 2); the combination item 1 + red flags group of items yielded 54% mean sensitivity and 95% mean specificity. Thereafter, we tested the algorithm including all the items and starting with item 1 that reached the greatest sensitivity. The second step was recognition of sensory trick, the item reaching the greatest specificity. In the absence of a sensory trick, including in the algorithm, the red flags group of items yielded the 84% mean sensitivity and 84% mean specificity (Table 2).

**Table 2:** Sensitivity and specificity of combination of clinical diagnostic items

	Sensitivity			Specificity			Average sensitivity (%)	Average specificity (%)
	Observer 1	Observer 2	Observer 3	Observer 1	Observer 2	Observer 3		
Repetitive, patterned head/neck movements/postures inducing head/neck deviation from neutral position (item 1) + sensory trick (item 2)	74.4% (32/43)	72.1% (31/43)	72.1% (31/43)	85.4% (35/41)	85.4% (35/41)	95.1% (39/41)	73.5	88.6
Repetitive, patterned head/neck movements/postures inducing head/neck deviation from neutral position (item 1) + red flags related to conditions mimicking dystonia (items 3–6)	58.1% (25/43)	51.2% (22/43)	51.2% (22/43)	95.1% (39/41)	100% (41/41)	90.1% (37/41)	53.5	95.1
Repetitive, patterned head/neck movements/postures inducing head/neck deviation from neutral position (item 1) + sensory trick (item 2) + red flags related to conditions mimicking dystonia (items 3–6)	86% (37/43)	81.4% (35/43)	83.7% (36/43)	80.5% (33/41)	85.4% (35/41)	85.4% (35/41)	83.7	83.8

Finally, the prior algorithm was further checked by omitting one of the red flags at a time. As reported in Table 3, the best combination of sensitivity and specificity was observed when item 6 (“capability to voluntarily suppress spasms”) was excluded.

**Table 3:** Sensitivity and specificity of clinical diagnostic items 1–6, omitting one of the red flags at a time (items 3–6)

	Sensitivity			Specificity			Average sensitivity (%)	Average specificity (%)
	Observer 1	Observer 2	Observer 3	Observer 1	Observer 2	Observer 3		
All items except item 3 (fixed head/neck deviation from neutral position)	86% (37/43)	81.4% (35/43)	83.7% (36/43)	70.7% (29/41)	80.5% (33/41)	75.6% (31/41)	83.7	76.2
All items except item 4 (focal weakness of neck muscles antagonizing the abnormal head/neck posture)	88.4% (38/43)	83.7% (36/43)	83.7% (36/43)	80.5% (33/41)	85.4% (35/41)	85.4% (35/41)	85.3	84.1
All items except item 5 (diplopia induced by voluntary correction of the abnormal head/neck posture)	86% (37/43)	81.4% (35/43)	83.7% (36/43)	65.8% (27/41)	75.6% (31/41)	80.5% (33/41)	83.7	74.6
All items except item 6 (ability to voluntarily suppress spasms)	97.6% (42/43)	95.3% (41/43)	95.3% (41/43)	75.6% (31/41)	85.4% (35/41)	80.5% (33/41)	96.1	81

## Discussion

Among the clinical items herein tested, items 1–4 (i.e., repetitive, patterned head/neck movements/postures inducing head/neck deviation from neutral position; sensory trick; tonic head/neck deviation from neutral position; and focal weakness of neck muscles antagonizing the abnormal head/neck posture) were evaluated for reliability and were found to have almost perfect inter-rater agreement. Items 5 and 6 (i.e., diplopia induced by voluntary correction of the abnormal head/neck posture; and ability to voluntarily suppress spasms) were not tested for reliability because they were assessed by a patient’s answer to a standardized question.

With regard to accuracy, the item “patterned, repetitive head/neck movements/postures inducing head deviation from neutral position” achieved very high sensitivity (98% on average), thus confirming the suggestion present in the 2013 definition of dystonia that assigns a crucial role to this item in diagnosing and differentiating CD from other neck movement disorders, like chorea and tremor [1]. Nevertheless, the 49% specificity indicated a high risk of misclassifying several cases. Neither sensory trick nor red flags alone provided satisfactory sensitivity and specificity.

Since these accuracy estimates were unsatisfactory, we tested whether combining the selected items improved diagnostic sensitivity/specificity. We observed that combining item 1 (the item that reached the greatest sensitivity) and sensory trick (the item that reached the greatest specificity) increased specificity to 89% but decreased sensitivity to 73%. This was probably because sensory trick is a feature largely specific for dystonia but is not present in about 20% of CD patients [4, 5]. It should be noticed that among control subjects, 4 patients with tic, 3 patients with myasthenia, and 1 patient with ocular torticollis reported that touching the neck was a sensory trick. In tic patients, we could not be sure that this was a true trick or the result of the voluntary spasm suppression characteristic of tics [20]. In the other controls, tactile stimulation/local compression of the muscle may have led to the alleviation of muscle weakness and improvement in neck position.

**Fig. 1:**
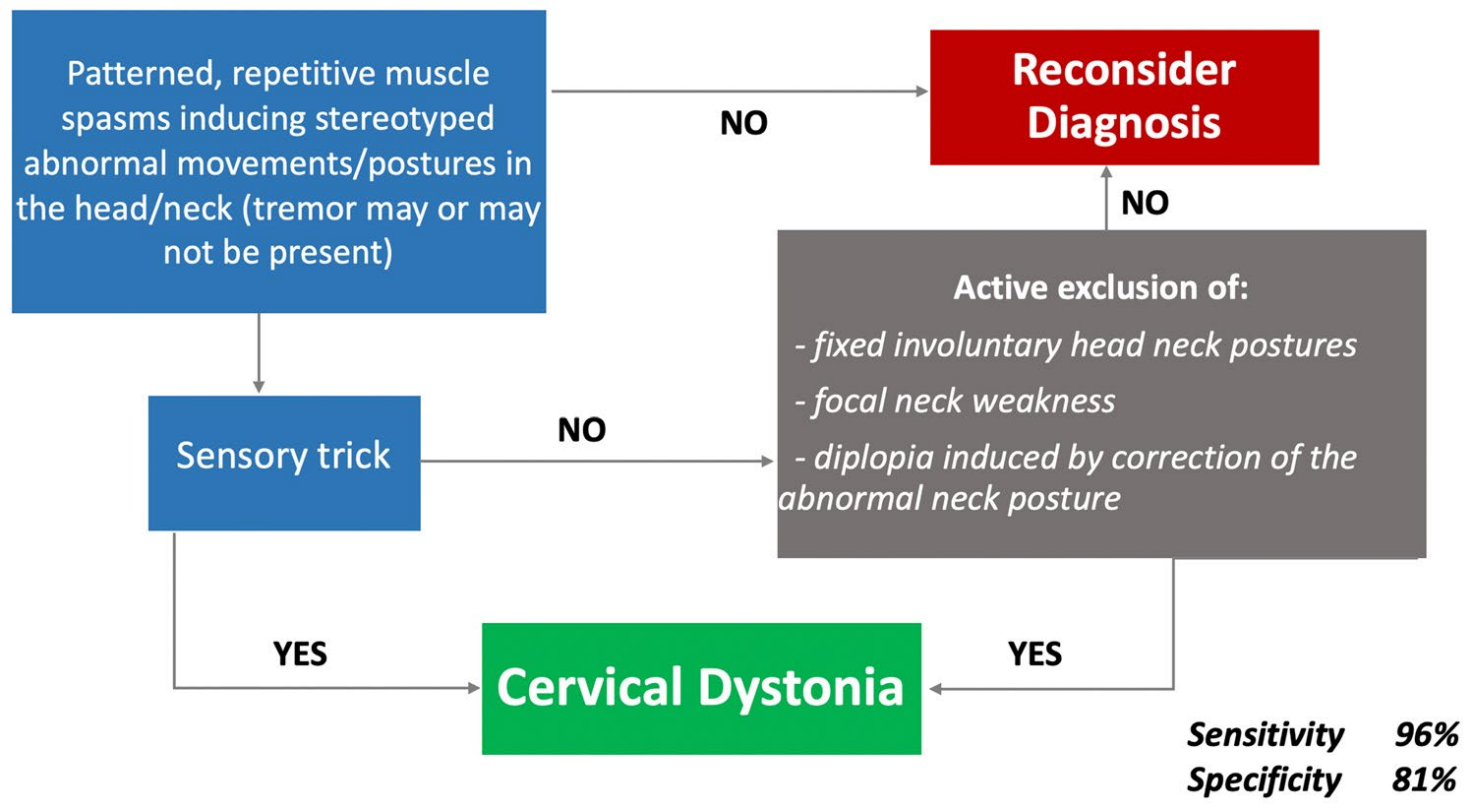
Proposed algorithm for the diagnosis of cervical dystonia

In the absence of a sensory trick, active exclusion of the red flags (items 3–6) yielded 84% mean sensitivity and 84% mean specificity. Finally, we tested shorter versions of the algorithm by excluding one of the red flags at a time. The best combination of sensitivity and specificity was obtained when the item “ability to mentally suppress spasms” was omitted. This is a feature that is potentially useful in distinguishing dystonia and tics, because tics are voluntarily suppressible while dystonia is not. The lack of utility of this item may be because voluntary suppressibility can be difficult to ascertain, and the result may depend closely on how the question is asked [27]. Some patients with CD may interpret the ability to voluntarily suppress symptoms in different ways. They may believe that “voluntary” includes compensatory movements that counteract dystonic movements or postures or they may believe that suppressibility can be partial. In fact, several CD patients reported voluntary suppressibility, which may reflect these differences of interpretation. Regardless of the explanation, the average 96% sensitivity and 81% specificity obtained with this algorithm (Fig. 1) means that it can correctly diagnose CD in more than 9/10 patients who have the condition and correctly identify 8/10 subjects who do not have the condition (Fig. 1).

Our study has several strengths. First, the validation procedure included patients with CD (whose demographic and clinical characteristics resembled those of patients reported in other published series), healthy controls, and subjects with a variety of neck disorders mimicking CD. Second, the standardized videotape protocol reproduced all major features seen during clinical examination. However, the present study also has some limitations. We did not evaluate whether incorporating the proposed guideline was better than providing only brief training without specific criteria to the raters. Nevertheless, there are several lines of evidence indicating that, in the absence of specific criteria, there is variability in the diagnostic approach of physicians, regardless of their expertise [28, 29]. Our aim was to provide a valid and practical guideline capable of reducing variability among physicians. There may also be variability in the interpretation of patients to answer standardized questions on video examination. Probably a live examination would provide better outcome than video examination. Likewise, specificity will probably be better in real life than in this sample where the number of mimics closely matched the number of cases. Finally, since all patients and evaluating physicians involved in this study were from the same country, the results of this study need to be confirmed in different patient and physician populations.

Despite these limitations, this study demonstrates two relevant points. First, an accurate diagnosis of CD is not possible if we refer only to the core clinical feature of CD as proposed in the 2013 revised classification of dystonia [1], i.e., “patterned and repetitive movements/postures in the head/neck” as well as to the combination of this item and sensory trick. Second, a higher diagnostic accuracy can be achieved if we also consider clinical features related to dystonia mimics that should be absent in dystonia. The diagnostic algorithm without the item “ability to voluntarily suppress spasms” was sensitive and specific enough to be proposed as a guideline for presumptive diagnosis of CD, though it needs to be further expanded and validated in a larger international sample.

## Supplementary Information

Below is the link to the electronic supplementary material.

Supplementary file1 (DOCX 13 KB)

Supplementary file2 Receiver operating characteristic curve displaying sensitivity and specificity and area under the curve for the diagnostic algorithm displayed in Figure 1. The arrow indicates the best combination of sensitivity and specificity discriminating patients with cervical dystonia from controls (PNG 150 KB)

## Data Availability

Data are available upon reasonable request.
