# Supplementary material for: Validation of a guideline to reduce variability in diagnosing cervical dystonia
Source: J Neurol. 2023 Feb 15;270(5):2606–12. doi: 10.1007/s00415-023-11585-6 (PMC10129917; doi:10.1007/s00415-023-11585-6)
Supplement: Supplementary file 1 — Supplementary file1 (DOCX 13 KB) [file 415_2023_11585_MOESM1_ESM.docx]

**Supplemental table 1.** Clinical aspects of cervical dystonia mimics included in the control group

| ***Cervical dystonia mimic*** | ***Clinical features differentiating the condition from cervical dystonia*** |
| --- | --- |
| Chorea | Non repetitive involuntary head movements |
| Isolated head tremor | Lack of dystonic posturing |
| Head tics | Ability to mentally suppress spasms |
| Orthopedic neck diseases (like atlanto-axial and shoulder subluxation or cervical vertebrae fracture) | Fixed involuntary head/neck deviation from neutral position |
| Rheumatologic neck diseases and posterior fossa tumors | Fixed involuntary head/neck deviation from neutral position |
| Lower motor neuron disease/myopathy/myasthenia gravis affecting neck muscles | Focal weakness of the neck muscles opposite the side of abnormal posture |
| Ocular torticollis | Diplopia caused by the voluntary correction of abnormal neck posture |
