# Supplementary figures and images for: Validation of a guideline to reduce variability in diagnosing cervical dystonia
Source: J Neurol. 2023 Feb 15;270(5):2606–12. doi: 10.1007/s00415-023-11585-6 (PMC10129917; doi:10.1007/s00415-023-11585-6)

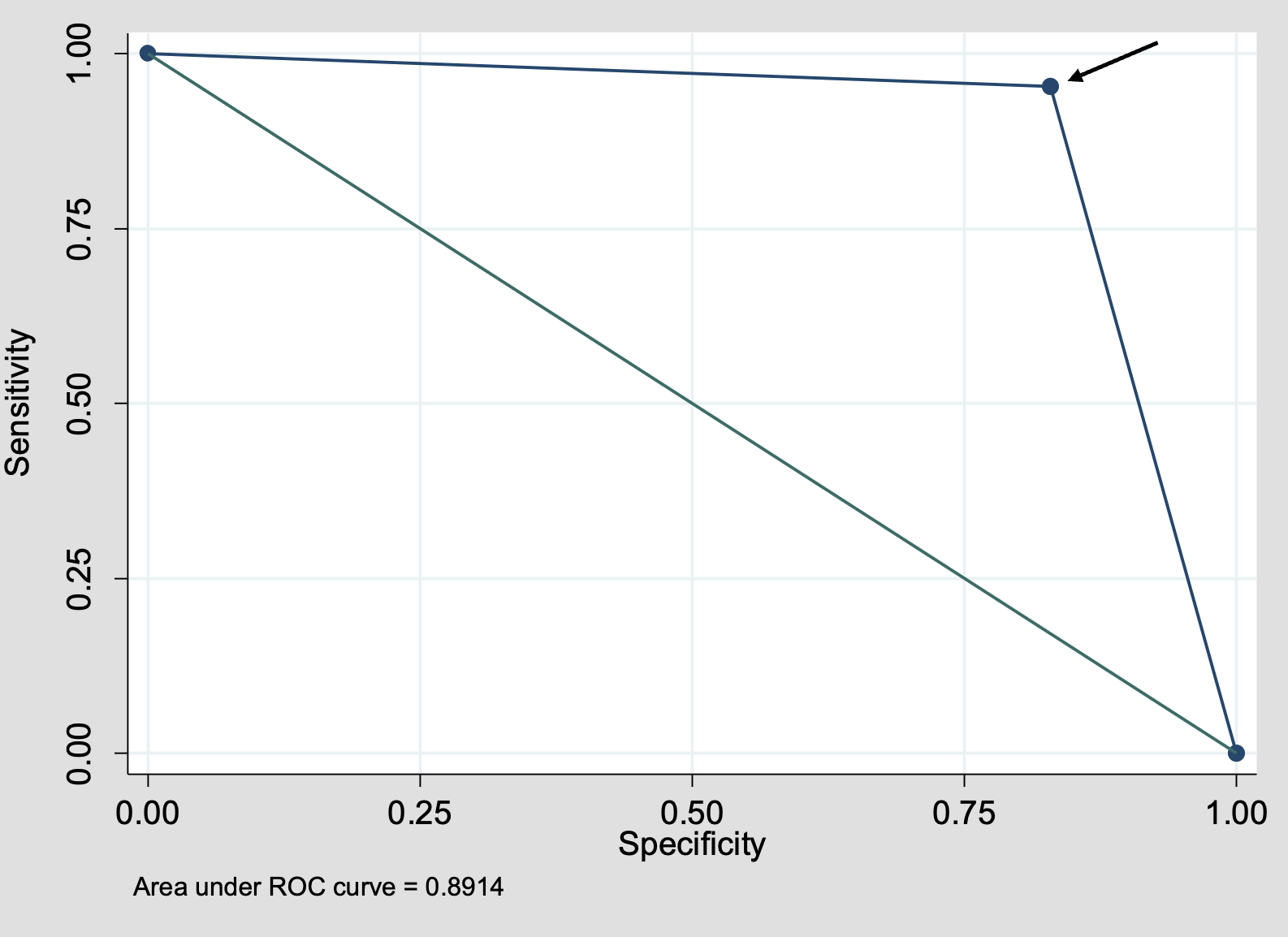

Supplement: Supplementary file 2 — Supplementary file2 Receiver operating characteristic curve displaying sensitivity and specificity and area under the curve for the diagnostic algorithm displayed in Figure 1. The arrow indicates the best combination of sensitivity and specificity discriminating patients with cervical dystonia from controls (PNG 150 KB) [file 415_2023_11585_MOESM2_ESM.png]
